# PD61 Thromboprophylaxis After Major Orthopedic Surgeries: Health Technology Assessment To Promote Access To Oral Anticoagulants

**DOI:** 10.1017/S0266462324003192

**Published:** 2025-01-07

**Authors:** Mayra Carvalho Ribeiro, Giovana Fernanda Santos Fidelis, Carlos Roberto Silveira Correa, Lucieni de Oliveira Conterno, Flávia de Oliveira Motta Maia

## Abstract

**Introduction:**

While implementing an evidence-based guideline for venous thromboembolism (VTE) prophylaxis in a Brazilian tertiary hospital, we identified an unmet need for patients undergoing major orthopedic surgery. The Brazilian Unified Health System (SUS) does not provide access to direct oral anticoagulants (DOACs) or enoxaparin. Therefore, an assessment of the efficacy, safety, and budgetary impact of these medications from a hospital perspective is warranted.

**Methods:**

Our Health Technology Assessment Center performed an overview of systematic reviews (SR) to compare the efficacy and safety of DOACs with enoxaparin. The Cochrane Library, Embase, and MEDLINE databases were searched in May 2023. The relative risks of symptomatic VTE, clinically relevant bleeding, and mortality were collected. The AMSTAR-2 tool was used to assess the methodological quality of included SRs. Treatment costs and estimates of the number of patients undergoing knee or hip arthroplasty were derived from historical institutional data.

**Results:**

Of the 32 SRs included in the analysis, seven performed a network meta-analysis. All SRs had at least one flaw in a critical methodological domain, mainly in not providing the list of excluded studies. Regarding mortality rates, most SRs did not detect any differences between the treatments. The risk of experiencing VTE was lower with DOACs in eight SRs for hip arthroplasty and in five SRs for knee arthroplasty. The risk of major bleeding was similar between the treatments in all but two SRs. Substituting enoxaparin for DOACs led to a cost reduction of BRL490 (USD98) per patient, which could save BRL29,890 (USD6,038) per year.

**Conclusions:**

Patients undergoing hip and knee arthroplasties are at high risk for the occurrence of VTE. Our overview of SRs showed that the efficacy and safety of DOACs are well recognized. DOACs reduce the risk of VTE, but to date patients in Brazil do not have access to these medicines through the SUS. By providing DOACs, hospitals could ensure adequate prophylaxis without increasing costs.

